# Mapping the landscape and exploring trends in macrophage-related research within non-small cell lung cancer: a comprehensive bibliometric analysis

**DOI:** 10.3389/fimmu.2024.1398166

**Published:** 2024-07-05

**Authors:** Yinxue Zhou, Tingyu Wu, Jiangxing Sun, Huanhuan Bi, Yuting Xiao, Hongmei Wang

**Affiliations:** ^1^ Department of Respiratory and Critical Care Medicine, The Affiliated Hospital of Qingdao University, Qingdao, China; ^2^ Department of Joint Surgery, The Affiliated Hospital of Qingdao University, Qingdao, China

**Keywords:** macrophage, NSCLC, bibliometric, citespace, VOSviewer

## Abstract

**Background:**

Macrophages play a pivotal role in the research landscape of non-small cell lung cancer (NSCLC), contributing significantly to understanding tumor progression, treatment resistance, and immunotherapy efficacy. In this study, we utilized bibliometric techniques to analyze shifts in research hotspots and trends within the field, while also forecasting future research directions. These insights aim to offer guidance for both clinical therapeutic interventions and foundational scientific inquiries.

**Methods:**

All publications were released between 1993 and 2023 and focus on research pertaining to macrophages in the field of NSCLC. The articles were identified from the Web of Science Core Collection and analyzed using VOSviewer 1.6.19, CiteSpace 6.2.R2, and Scimago Graphica 1.0.35.

**Result:**

A total of 361 articles authored by 3,072 researchers from 48 countries were included in the analysis. TAMs have gained increasing attention for their role in NSCLC development and as potential therapeutic targets. Modulating TAM behavior may offer avenues to suppress tumor growth and drug resistance, improving patient outcomes. International collaboration, particularly between China and the United States, accelerates progress in NSCLC research, benefiting patients worldwide. The research hotspot revolves around understanding the role of macrophages in immunotherapy, focusing on their contribution to tumor progression, therapeutic resistance, and potential as therapeutic targets in NSCLC.

**Conclusions:**

The therapeutic significance of macrophages in the field of NSCLC is gaining increasing attention and recognition, highlighting their potential as key players in the development of novel treatment strategies. Future research will focus on understanding TAM molecular mechanisms, interactions with immune cells, and exploring novel therapies, with the aim of improving NSCLC treatment outcomes.

## 1 Introduction

NSCLC constitutes the majority of lung cancer cases globally, with smoking as a primary risk factor (1). However, a significant proportion of NSCLC cases occur in non-smokers, indicating the multifactorial nature of the disease. Additionally, NSCLC encompasses various histological subtypes, including adenocarcinoma, squamous cell carcinoma, and large cell carcinoma, each with distinct clinical characteristics and treatment approaches (2). Standard therapies include surgical resection, chemotherapy, radiation therapy, and targeted therapies directed against specific oncogenic drivers such as EGFR mutations or ALK rearrangements (3, 4). Macrophages, particularly tumor-associated macrophages (TAMs), play crucial roles in NSCLC progression and treatment response. Within the tumor microenvironment, TAMs exhibit diverse functions that impact tumor behavior, including immune modulation, extracellular matrix remodeling, and angiogenesis (5–7). The resistance of NSCLC to EGFR-TKI therapy can be mediated by activating the PI3K/AKT pathway, and the enrichment of TAMs is also associated with resistance to immunotherapy (8, 9). Additionally, the accumulation of senescent macrophages in the alveoli promotes early lung cancer by upregulating the expression of P16 and inhibiting the cytotoxic T cell response (10). Targeted therapy against macrophages can facilitate communication between IFNγ-secreting lymphocytes and IL12-producing dendritic cells to enhance the antitumoral response (11). Therefore, the abundance, polarization states, and functional diversity of TAMs influence NSCLC prognosis and treatment outcomes (12).

Bibliometrics is indispensable in the field of medicine, overcoming the constraints of traditional literature reviews. By harnessing mathematical and statistical methods, it offers a quantitative assessment of research domains, revealing patterns, and predicting emerging areas of interest (13, 14). This approach not only provides a comprehensive understanding of the current state of medical knowledge but also aids in identifying gaps in research and guiding future inquiries. Although bibliometric analysis has been applied in fields such as immune checkpoint inhibitors and radiotherapy for NSCLC (15, 16), no comprehensive study has yet focused on the role of macrophages in NSCLC.

Addressing this gap, our study conducts a detailed bibliometric analysis specifically targeting macrophage research within NSCLC. Employing advanced tools such as VOSviewer and CiteSpace, we meticulously analyzed crucial information extracted from the literature, encompassing details regarding authors, journals, countries of origin, citations, and subsequently generated scientific visualizations (17). Our investigation delved into the dynamic evolution of research hotspots within this domain and elucidated the changing trends in scholarly pursuits over time. Moreover, we extrapolated potential trajectories for future research endeavors within this field, providing valuable insights for advancing our understanding of NSCLC and macrophage-related therapeutics.

## 2 Materials and methods

### 2.1 Data sources and search strategies

We selected the Science Citation Index Expanded (SCI-EXPANDED) and Social Sciences Citation Index (SSCI) from the Web of Science Core Collection for bibliometric analysis, acknowledging its wide-ranging content, comprehensive nature, and prompt data updates. Unlike commonly used databases such as PubMed and Scopus, Web of Science has the broadest coverage, including the oldest literature dating back to 1900. Its earliest publications can be traced back to 1900, surpassing the retrieval capabilities of PubMed and Scopus, which can only retrieve documents dating back to 1950 and 1966, respectively (18). Moreover, researchers have compared the accuracy of journal categorization and found that, in comparison to Scopus, WoS provides more precise journal categorization (19, 20). Furthermore, research into document types has shown that WoS has a 7% lower error rate in document type tagging compared to Scopus. Due to the fact that the initial literature pertaining to this study was issued in 1993, publications searched between January 1, 1993, and December 31, 2023, have been included in this study. The search terms were presented as follows: TS=((Macrophages or Bone Marrow-Derived Macrophages or Bone Marrow Derived Macrophages or Bone Marrow-Derived Macrophage or Macrophage, Bone Marrow-Derived or Macrophages, Bone Marrow-Derived or Monocyte-Derived Macrophages or Monocyte Derived Macrophages or Macrophage or Macrophages, Monocyte-Derived or Macrophage, Monocyte-Derived or Macrophages, Monocyte Derived or Monocyte-Derived Macrophage) AND (Carcinoma, Non-Small-Cell Lung or Carcinoma, Non-Small Cell Lung or Carcinomas, Non-Small-Cell Lung or Lung Carcinoma, Non-Small-Cell or Lung Carcinomas, Non-Small-Cell or Non-Small-Cell Lung Carcinomas or Non-Small-Cell Lung Carcinoma or Non-Small Cell Lung Carcinoma or Carcinoma, Non-Small Cell Lung or Non-Small Cell Lung Carcinoma or Non-Small Cell Lung Cancer or Nonsmall Cell Lung Cancer)). In conclusion, 11,819 documents were retrieved, encompassing various document subtypes such as articles, conference papers, and reviews.

### 2.2 Data collection and cleaning

**Figure 1:**
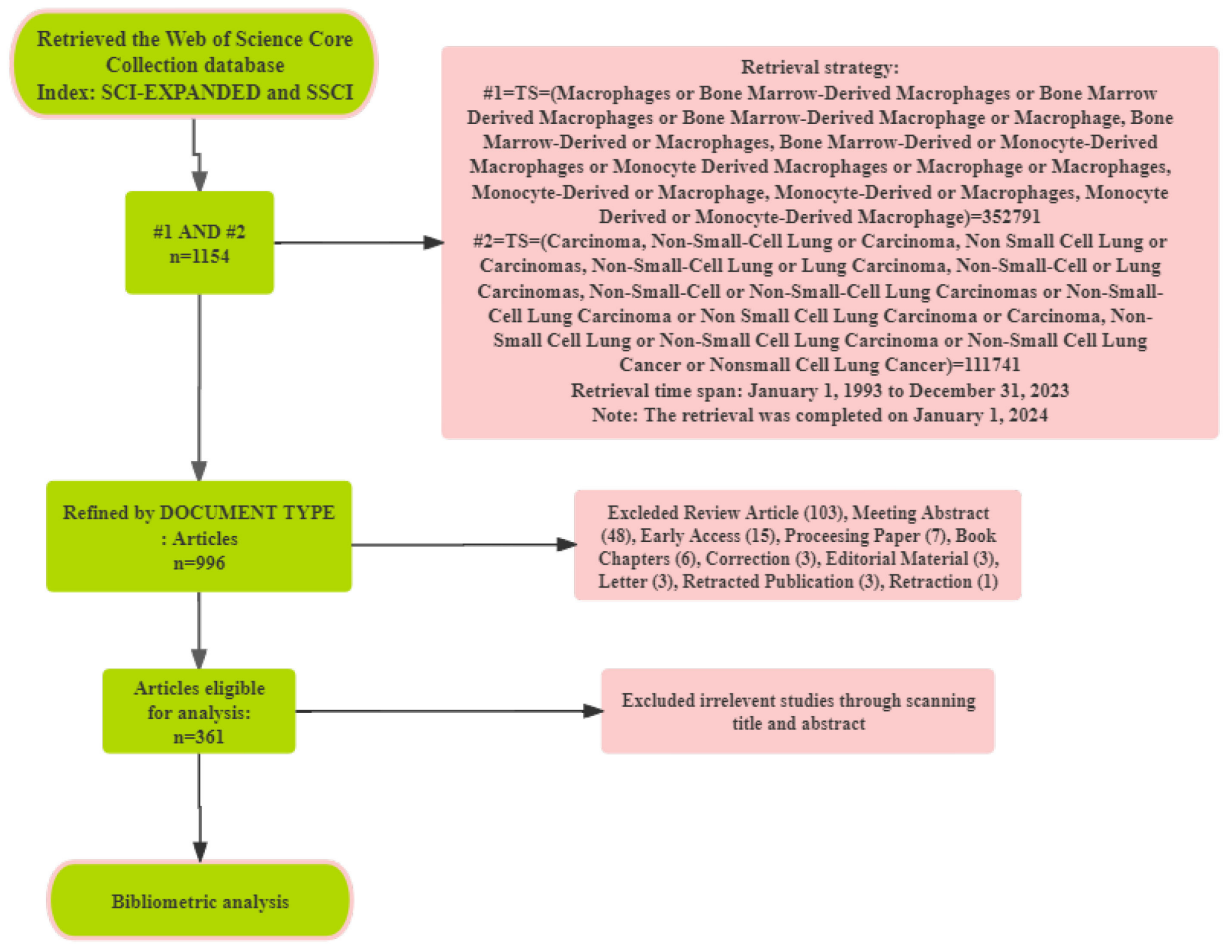
Flowchart of literature screening in this study.

The information obtained after acquiring the raw data includes the quantity and citation frequency of the literature, as well as details such as countries and regions, publication years, institutions, authors, references, journals, and keywords. All data is sourced from open databases. Despite potential biases in the data due to factors like identical abbreviations for different authors and multiple versions of cited literature, we have taken rigorous measures to enhance reliability. Multiple researchers conducted evaluations at critical stages, merged synonymous terms, corrected spelling errors, and employed other methods to minimize potential inaccuracies. Review papers provide a broad understanding of a research field, helping researchers quickly grasp the current status and historical development. However, research papers, which contain the latest data and original findings, can more directly and rapidly reflect the newest trends and directions in scientific research. Therefore, only articles were ultimately included as the subjects for visualization analysis. Moreover, during the search process, the use of search terms may yield some articles that are not entirely relevant to the research topic. This occurs because search term matches do not always fully reflect the specific content and focus of the articles. This underscores the importance of meticulous review and screening by multiple authors when conducting bibliometric analysis. To ensure that the included literature is highly relevant to the research topic, it is essential to read abstracts, introductions, and even full texts, thereby enhancing the accuracy and reliability of the research results. As a result, irrelevant articles were removed, and 361 documents were included in our study. The detailed screening process is illustrated in **Figure 1**. Finally, the data underwent analysis using CiteSpace and VOSviewer, ensuring a comprehensive and reliable foundation for our research.

### 2.3 Bibliometric analysis

Bibliometrics is a field of study that involves the quantitative analysis of scholarly publications and their patterns of publication, citation, and co-authorship. Its primary goal is to provide a systematic and objective framework for evaluating the impact, influence, and trends within academic literature. By examining citation networks, publication frequencies, and collaboration patterns, bibliometrics helps researchers and institutions gain insights into the scholarly communication landscape.

CiteSpace and VOSviewer are two prominent tools in the realm of bibliometrics. CiteSpace specializes in visualizing and analyzing citation networks, enabling researchers to identify key papers, influential authors, and emerging research trends (21). It facilitates the exploration of intellectual structures within a knowledge domain. On the other hand, VOSviewer focuses on visualizing co-authorship and co-citation networks, providing a comprehensive overview of collaborative relationships and thematic clusters in scientific literature (22). Both tools play crucial roles in uncovering hidden patterns, facilitating literature reviews, and supporting evidence-based decision-making in academic research. Overall, bibliometrics, along with tools like CiteSpace and VOSviewer, contributes significantly to enhancing our understanding of scholarly landscapes and shaping the direction of future research endeavors. Overall, these tools offer a diverse and impartial view of the advancements in research on macrophages in the context of non-small cell lung cancer.

## 3 Result

### 3.1 An overview of publications on macrophage in NSCLC

Based on the search strategy, between 1993 and 2023, a total of 361 publications on targeting macrophages to inhibit the growth of non-small cell lung cancer were cited 12,664 times, with an average of 34.7 citations per item. Excluding self-citations, the count stands at 12,496. The research field holds an H-index of 60, indicating significant academic impact and research activity (23).

**Figure 2:**
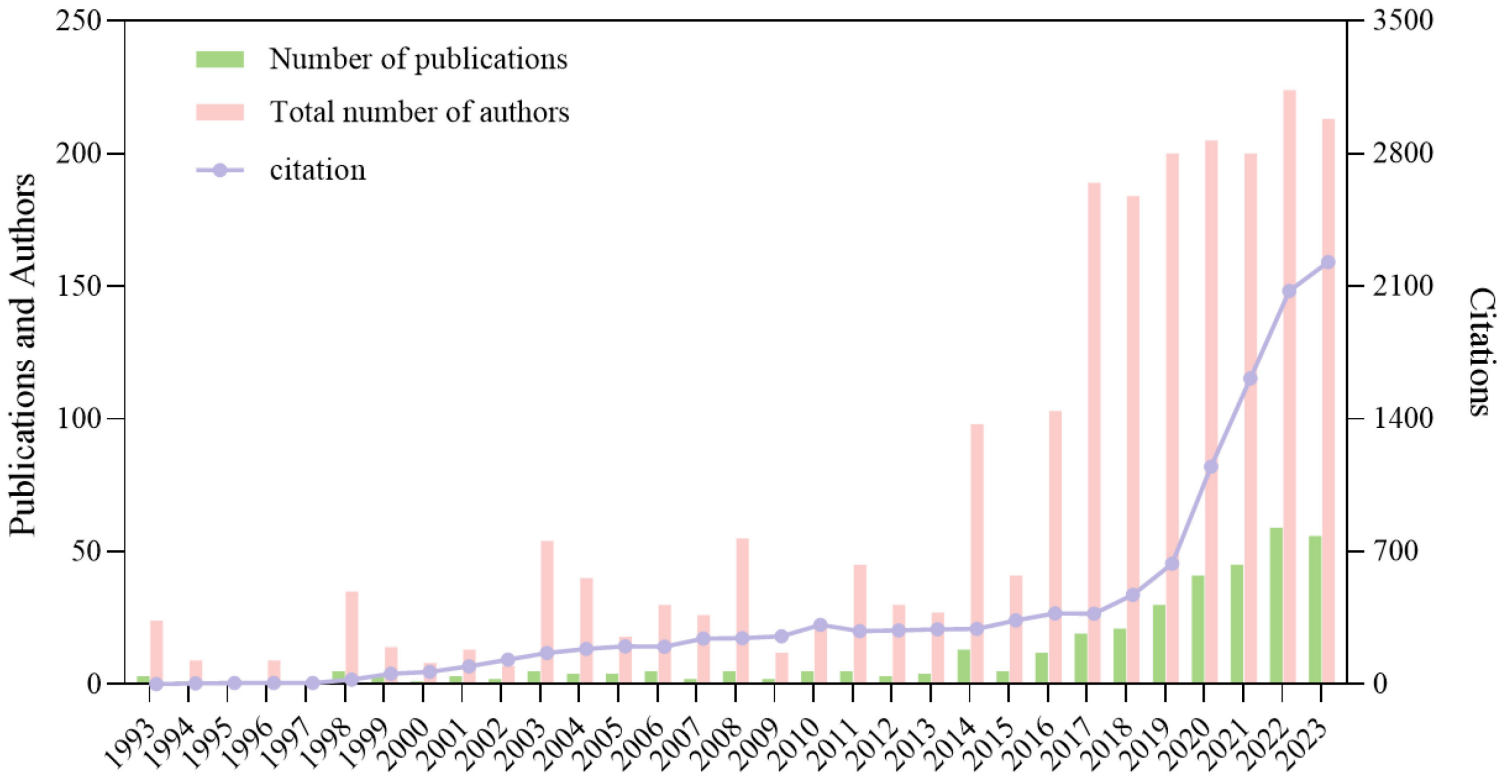
Global trend of annual publications, authors and total citations on macrophage for NSCLC.

Further analysis, as visualized in **Figure 2**, reveals a marked growth in research activity over the past thirty years. The graph shows an upward trend in both the number of publications and the total number of authors contributing to the field, with citations increasing substantially in the last few years. This pattern suggests an escalating engagement with and recognition of the importance of macrophage-targeted therapies in non-small cell lung cancer research.

### 3.2 Analysis of authors

Price D. S, a historian of science in the United States, found that the quantity of prolific authors responsible for half of all published papers equals the square root of the total author count (Formula 1) (24). Formula 2 offers a means to compute the minimum number of publications required for an author to be recognized as a core contributor in a particular field. Here, n(x) represents the number of authors who have written x papers, n is the total number of authors, I = *n_max_* is the number of papers published by the most prolific author in the field, and m is the minimum number of publications required to be considered a core author. In the field of non-small cell lung cancer, over 3,000 authors have actively engaged in macrophage research. Using VOSviewer analysis, the *n_max_* value is determined to be 5, meaning that authors who have published more than two papers are considered core contributors. Thus, out of these 3,000 authors, 188 are recognized as core authors.


(1) $$\sum_{m+1}^{I}n(x)=\sqrt{n}$$

(2) $$m=0.749\times \sqrt{n_{max}}$$

**Figure 3:**
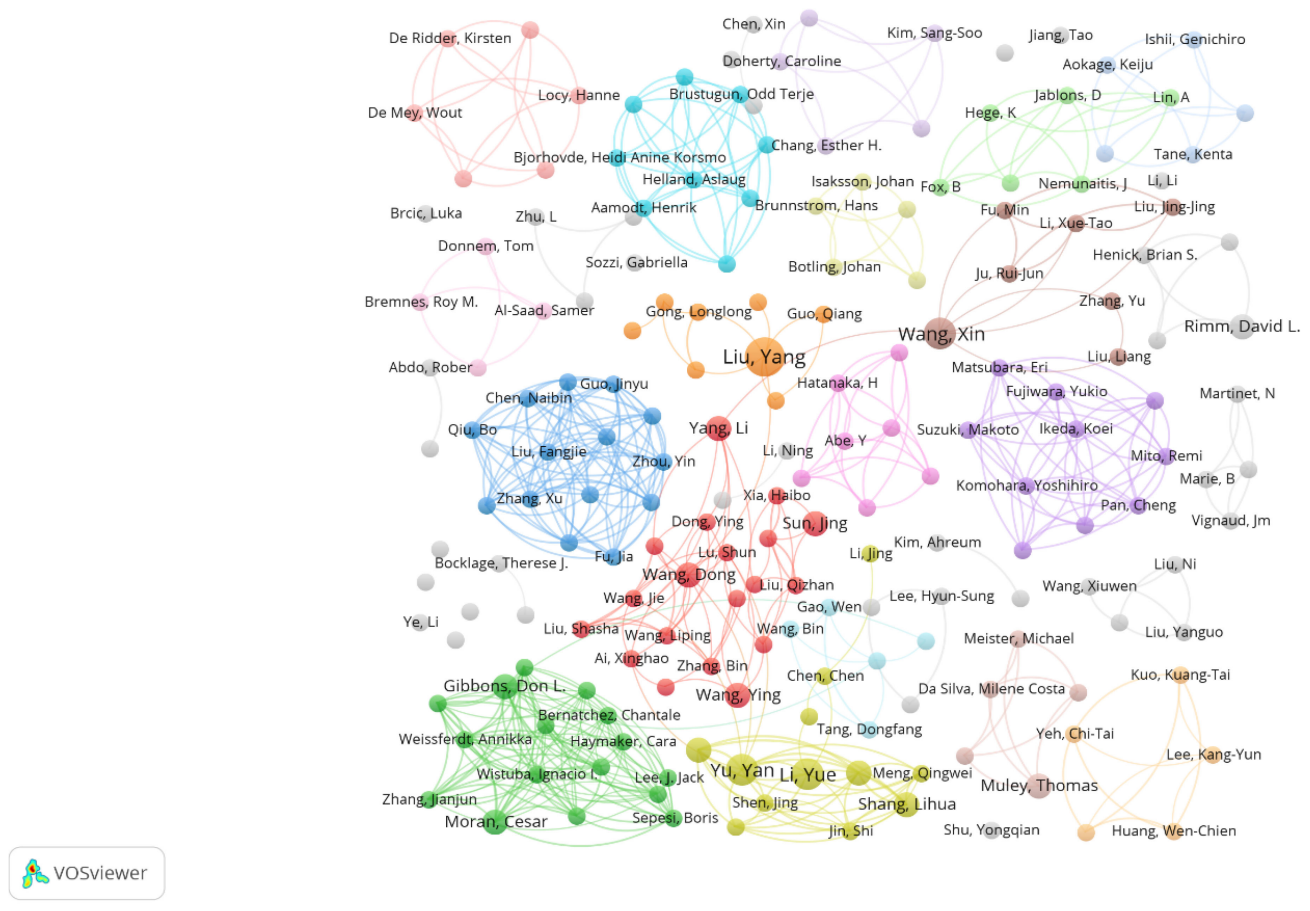
Network visualization map of author co-authorship analysis. The size of the points in the graph represents the magnitude of the authors’ contributions, while the lines depict collaborations among different authors. The various colors denote distinct collaborative clusters formed spontaneously among the authors.

The top ten most prolific core authors, as illustrated in **Table 1**, hail from various countries. Liu, Y from Wake Forest University School of Medicine has contributed the highest number of articles. Notably, researchers from China constitute the largest group, totaling eight individuals, while the remaining two are from the United States. Additionally, Rimm, D. I. from Yale School of Medicine exhibits a high AC value, reflecting his significant academic impact in this field. Additionally, **Figure 3** presents a network visualization of co-authored works, where the size of each node represents the contribution level of each author, and the lines denote the connections between different authors. Some prolific authors, such as Yu Yan, Li Yue, and Shang Lihua, form distinct clusters with tight interconnections, whereas their interactions with other clusters are less frequent.

**Table 1:** The 10 most productive authors in the field of macrophage for NSCLC.

Rank	Author	Affiliation	Documents	Citations	TLS	AC
1	Liu, Y	Wake Forest University School of Medicine	5	36	1	7.2
2	Yu, Y	Harbin Medical University Cancer Hospital	4	345	12	86.25
3	Wang, X	University of Rochester School of Medicine and Dentistry	4	94	1	23.5
4	Li, Y	Harbin Medical University Cancer Hospital	4	199	8	49.75
5	Yang, L	Biotherapy Center, The First Affiliated Hospital of Zhengzhou University	3	150	2	50
6	Wang, Y	Weifang Medical University	3	62	0	20.67
7	Wang, D	Third Military Medical University	3	205	3	68.33
8	Sun, J	Nanjing Medical University	3	176	0	58.67
9	Shang, L	Harbin Medical University Cancer Hospital	3	239	11	79.67
10	Rimm, D. I.	Yale School of Medicine	3	273	0	91

TLS, Total link strength; AC, average citations.

### 3.3 Analysis of journals

**Figure 4:**
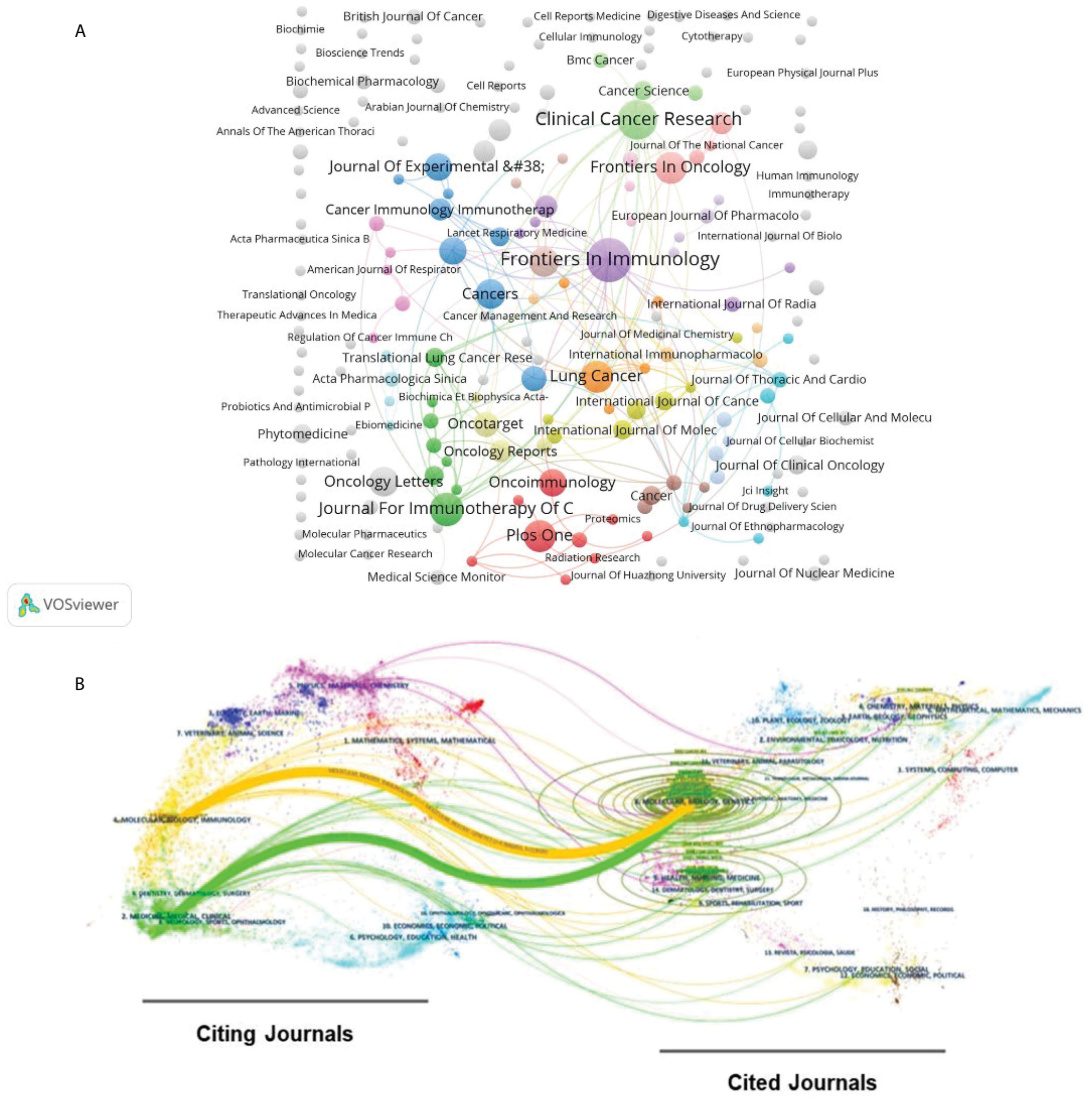
**(A)** Network visualization map of Citing Journals. The size of the circles represents the volume of publications. **(B)** Dual-map overlay of journals produced by CiteSpace. The citing journals are located on the left, and the cited journals are located on the right. The color paths (one main orange and one main green reference paths) represent the cited relationship.

VOSviewer and CiteSpace are utilized for the analysis of cited and citing references, ultimately identifying the journals with the highest publication count and the most cited journals in the field (**Table 2**). Among them, *frontiers in immunology, clinical cancer research* and *journal for immunotherapy of cancer* stands out as the top three journals with the highest publication counts. Correspondingly, in the network visualization of journals shown in **Figure 4A**, the larger nodes effectively illustrate the high output of these journals. Within the top ten most prolific journals, *clinical cancer research* holds the distinction of having the highest impact factor. Additionally, *cancer research clinical cancer research* and *New England Journal of Medicine* represents the top three journals with the highest citation counts, all of which are classified in the first quartile according to JCR partitions.

**Table 2:** The 10 most productive authors and the top 10 authors with most citations in the field of macrophage for NSCLC.

Rank	Source	Documents	Citations	IF	JCR	Source	Citations	IF	JCR
1	frontiers in immunology	15	391	7.3	Q1	cancer research	565	11.2	Q1
2	clinical cancer research	12	1234	11.5	Q1	clinical cancer research	435	11.5	Q1
3	journal for immunotherapy of cancer	9	339	10.9	Q1	New England Journal of Medicine	389	158.5	Q1
4	plos one	8	302	3.7	Q2	Journal of Clinical Oncology	301	45.4	Q1
5	lung cancer	8	259	5.3	Q1	nature	342	64.8	Q1
6	frontiers in oncology	8	37	4.7	Q2	cell	329	64.5	Q1
7	cancer research	7	7	11.2	Q1	Journal of Immunology	285	4.4	Q2
8	cancers	7	99	5.2	Q1	Proceedings of the National Academy of Sciences of the United States of America	248	11.1	Q1
9	oncology letters	7	62	2.9	Q3	Journal of Thoracic Oncology	232	20.4	Q1
10	journal of experimental & clinical cancer research	6	367	11.3	Q1	plos one	218	3.7	Q2

The journal overlay graph effectively illustrates the relationship between journals and their cited counterparts (**Figure 4B**). It highlights that papers published in journal of *Molecular, Biology and Immunology* and journal of *Medicine, Medical and Clinical* are frequently cited in papers published in journal of *Molecular, Biology and Genetics*.

### 3.4 Analysis of countries

**Figure 5:**
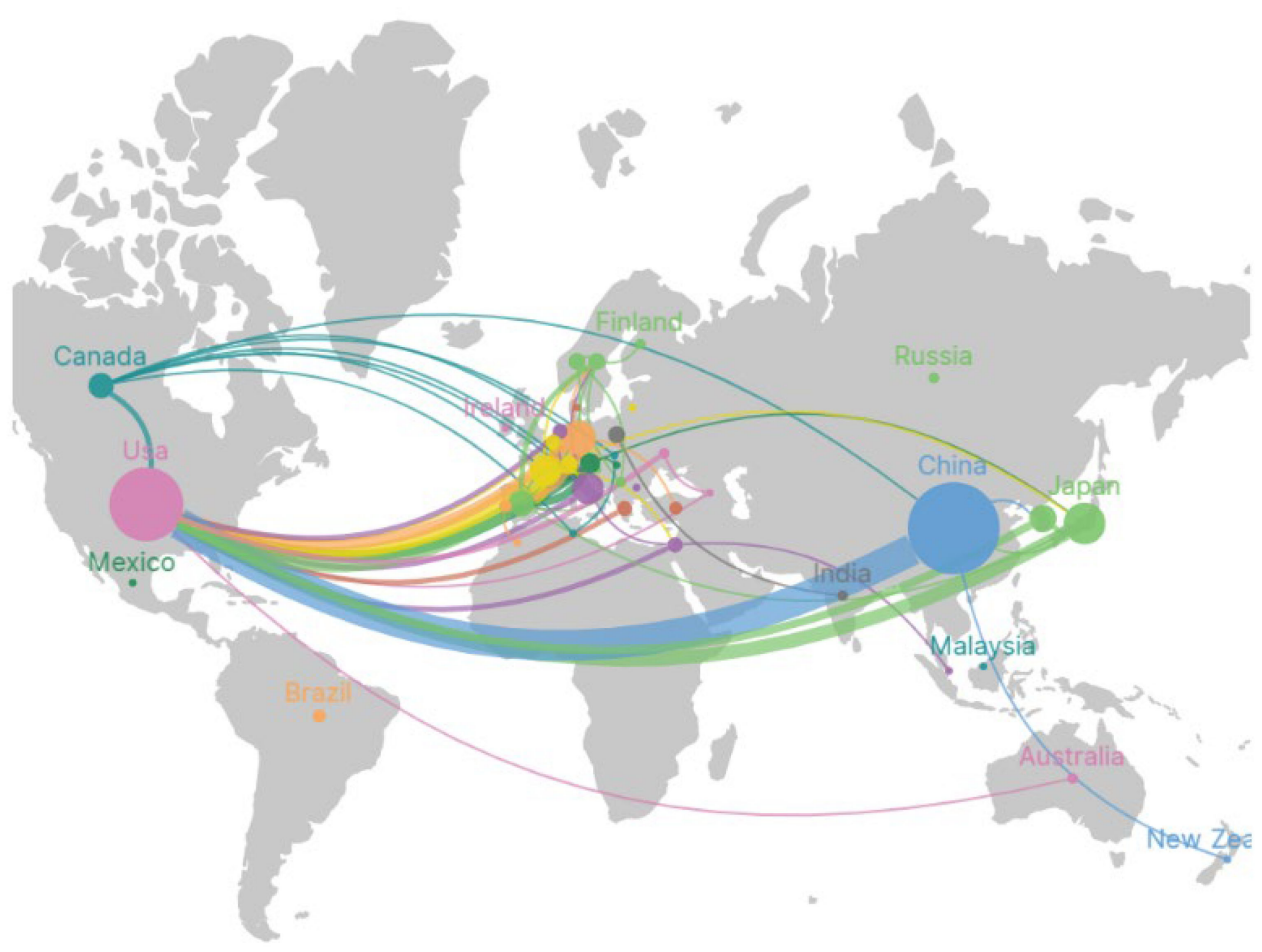
Geographic distribution map based on publications from different countries/regions: different colors represent different countries, while the lines depict collaborations between countries. Thicker lines indicate more extensive collaboration between nations, and larger nodes signify a higher volume of publications from the respective countries.

Moreover, through VOSviewer analysis, it was found that the eligible literature was authored by individuals from 48 different countries. Scimago Graphica was employed to scrutinize collaboration patterns among publications from various nations, as depicted in **Figure 5**. In this visualization, the node sizes correspond to the publication quantities of different countries, while the connecting lines denote collaborations between nations, with thicker lines indicating more frequent collaborations. Additionally, the colors of the nodes represent clusters of countries with similar characteristics. **Figure 5** reveals the uneven distribution of publications among different countries. The node representing China is the largest, and the thickest line connects it to the similarly large node representing the United States. This indicates more frequent collaborations between countries with higher publication outputs.

**Table 3** presents a breakdown of the top five countries contributing to research on macrophages in the realm of non-small cell therapy. China takes the lead with an impressive count of 146 published articles, followed by the United States, Japan, Germany, and France. Despite a lower publication volume in the United States compared to China, it secures the top spot in the average citation per paper. This underscores the substantial academic influence of the articles originating from the United States in this specific field of study.

**Table 3:** Top 5 countries in the feld of macrophage for NSCLC.

Rank	Country	Publications	Citations	AC
1	China	146	3772	25.84
2	USA	94	6008	63.91
3	Japan	30	921	30.7
4	Germany	20	698	34.9
5	France	17	911	53.59

### 3.5 Analysis of cited reference and reference burst

Using VOSviewer, the top ten most cited articles were analyzed, as depicted in **Table 4**. Among them, three articles were published in the Cancer Research journal, while two were published in Nature. The three most highly cited articles were authored by Huang, M for the title “ Non-small cell lung cancer cyclooxygenase-2-dependent regulation of cytokine balance in lymphocytes and macrophages: Up-regulation of interleukin 10 and down-regulation of interleukin 12 production” (25), Chang, T. H for the title “ Induction of differentiation and apoptosis by ligands of peroxisome proliferator-activated receptor γ in non-small cell lung cancer” (26) and Disis, M. L. for the title “ Generation of T-cell immunity to the HER-2/neu protein after active immunization with HER-2/neu peptide-based vaccines” (27). Given the positive correlation between total citation count and publication duration, to mitigate the influence of time, we calculated the annual average total citation count. The results reveal that the article titled “Tissue-resident macrophages provide a pro-tumorigenic niche to early NSCLC cells” (5) published in Nature in 2021, leads significantly with an annual average citation count of 71, followed by the article published in Clinical Research, titled “Antibody-Fc/FcR Interaction on Macrophages as a Mechanism for Hyperprogressive Disease in Non-small Cell Lung Cancer Subsequent to PD-1/PD-L1 Blockade” (28) which holds the second position with an annual average citation count of 55.4.

**Table 4:** Top 10 highly cited articles in the field of macrophage for NSCLC.

Rank	Paper title	Author	Year	Journal	TC	TC per year
1	Non-small cell lung cancer cyclooxygenase-2-dependent regulation of cytokine balance in lymphocytes and macrophages: Up-regulation of interleukin 10 and down-regulation of interleukin 12 production	Huang, M.	1998	Cancer Research	409	15.73
2	Induction of differentiation and apoptosis by ligands of peroxisome proliferator-activated receptor γ in non-small cell lung cancer	Chang, T. H	2000	Cancer Research	370	15.42
3	Generation of T-cell immunity to the HER-2/neu protein after active immunization with HER-2/neu peptide-based vaccines	Disis, M. L.	2002	Nature	338	15.36
4	Antibody-Fc/FcR Interaction on Macrophages as a Mechanism for Hyperprogressive Disease in Non-small Cell Lung Cancer Subsequent to PD-1/PD-L1 Blockade	Lo Russo, G.	2019	Clinical Cancer Research	277	55.4
5	Tissue-resident macrophages provide a pro-tumorigenic niche to early NSCLC cells	Casanova-Acebes, M.	2021	Nature	213	71
6	Her-2/neu-derived peptides are tumor-associated antigens expressed by human renal cell and colon carcinoma lines and are recognized by *in vitro* induced specific cytotoxic T lymphocytes	Brossart, P.	1998	Cancer Research	211	8.12
7	Low-Dose Apatinib Optimizes Tumor Microenvironment and Potentiates Antitumor Effect of PD-1/PD-L1 Blockade in Lung Cancer	Zhao, S.	2019	Cancer Immunology Research	202	40.4
8	Glucose transporters and FDG uptake in untreated primary human non-small cell lung cancer	Brown, R. S.	1999	Journal of Nuclear Medicine	197	7.88
9	Nicotine inactivation of the proapoptotic function of Bax through phosphorylation	Xin, M. G.	2005	Journal of Biological Chemistr	191	10.05
10	Astragalus-based Chinese herbs and platinum-based chemotherapy for advanced non-small-cell lung cancer: meta-analysis of randomized trials	McCulloch, M.	2006	Journal of clinical oncology	189	23.63

TC, total citations.

**Figure 6:**
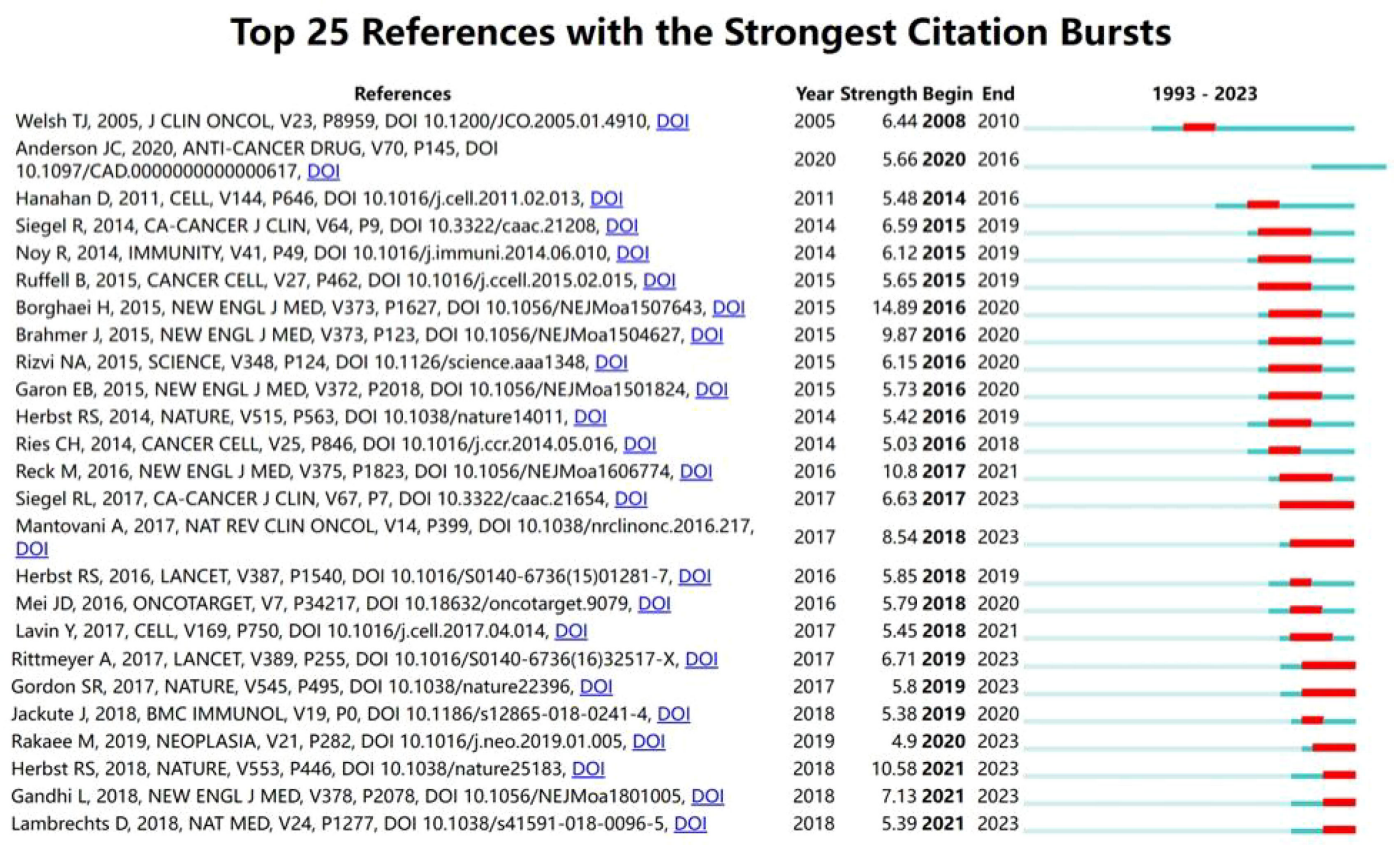
Top 25 references with strongest publication bursts: the light blue bars represent the duration from the start of the research to the end date (1993-2023), the dark blue indicates the time of publication, and the red signifies the burst period.

Additionally, by using CiteSpace, we conducted a citation burst analysis of the top 25 referenced works in this field (**Figure 6**). Burst analysis identifies rapid increases in specific words or phrases within a research area, aiding researchers in identifying development trends and hotspots in the field (29). These 25 papers experienced citation bursts between 2008 and 2023, with six of them continuing to show burst activity up until the end of the study period. It is noteworthy that the citation surge of these articles began after 2016, indicating a further increase in attention to research in this field since that year.

### 3.6 Analysis of keywords

**Figure 7:**
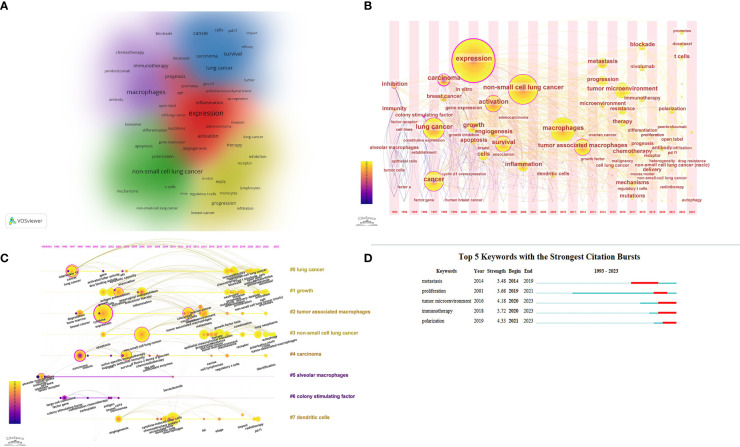
Keywords in the field of macrophage for NSCLC. **(A)** Co-occurrence of high-frequency keywords; The size of each character represents the frequency of occurrence, with different colors indicating distinct clusters. **(B)** The timezone view of high-frequency keywords; The size of each circle represents the frequency of keyword occurrences, while the lines indicate co-occurrence relationships between keywords. **(C)** The timeline view of high-frequency keywords; Circle size represents keyword frequency, lines indicate co-occurrence, and cluster labels are on the right. **(D)** Top 10 keywords with the strongest citation bursts; the light blue bars represent the duration from the start of the research to the end date (1993-2023), the dark blue indicates the occurrence time of keywords, and the red signifies the burst period.

According to Price’s theorem, keywords that appear more than eight times are identified as core keywords. A total of 72 core keywords have been categorized into 5 clusters, as illustrated in the **Figure 7A**. Using VOSviewer, core keywords were grouped into clusters with similar characteristics, as shown in **Figure 7A**, each represented by a different color. The red cluster, including activation, adenocarcinoma, and angiogenesis, describes the molecular and cellular biology of tumors, involving gene expression and cell behavior. The green cluster, with keywords like apoptosis, delivery, and dendritic cells, focuses on immunology research and experimental models, covering cell apoptosis, gene expression, and immune responses. The blue cluster, including cancer, carcinoma, and cells, encompasses a wide range of cancer research topics, reflecting clinical and general cancer studies. The yellow cluster, with keywords like *in-vitro*, infiltration, and inhibition, centers on specific cancers (such as lung cancer) and their treatments, emphasizing *in vitro* experiments, tumor progression, and immune cell infiltration. The purple cluster, including antibody, biomarker, and blockade, focuses on therapeutic approaches and drugs, highlighting different treatment methods and their applications in cancer therapy.

We utilized CiteSpace to construct timeline and time-zone maps of co-cited keywords, which cluster keywords while also considering the temporal dimension. These aids enable us to explore the evolutionary trajectory of research hotspots within the field, as depicted in **Figures 7B, C**. Early frequently occurring keywords included “expression,” “growth,” “angiogenesis,” and “survival” from 1999 to 2003, focusing on the role of macrophages in non-small cell lung cancer (NSCLC). After 2014, the keywords shifted to “metastasis,” “tumor microenvironment,” “resistance,” “chemotherapy,” and “immunotherapy.” This shift reflects a significant change towards studying the complex interactions between macrophages and the dynamic changes within the tumor microenvironment. A substantial amount of attention is now directed towards exploring the potential of targeting macrophage interventions in conjunction with emerging immunotherapy, radiotherapy, and chemotherapy approaches.

The citation burst analysis of keywords illustrated in **Figure 7D** unveils peaks of scholarly attention towards pivotal topics over time, delineating a shift in research focus. As shown in the figure, the keywords currently experiencing citation bursts include “tumor microenvironment,” “immunotherapy,” and “polarization.”

## 4 Discussion

### 4.1 Current research patterns in macrophage studies in NSCLC

Through systematic analysis of annual publications, citations, and authorship concerning macrophages in NSCLC, we have discerned the evolving research trends in this field. Overall, prior to 2017, research on macrophages in NSCLC remained relatively stable, with modest engagement and publication output. However, a significant shift occurred in 2017 with groundbreaking research, such as that published in Nature by scholars like Gordon, S. R, whose study that year was notably highly cited. Their findings demonstrated that drugs targeting the PD-1-PD-L1 interaction, known for their efficacy in immunotherapy, also affect tumor growth by modulating macrophage phagocytic activity (30). This discovery sparked widespread interest in macrophages as potential therapeutic targets in cancer immunotherapy, leading to a noticeable annual increase in publications, citations, and active participation within the NSCLC research community post-2017. Among the top ten authors in publication output, Professor Rimm D. I., who holds the highest average citation ranking, stands out, especially for studies on the relationship between macrophage expression of PD-L1 and T cell infiltration, indicating a potential future focal trend in the field (31). Through meticulous scrutiny of journal publications and their citation rates, it’s evident that five of the top ten premier journals in this field are situated in the Q1 JCR region. Moreover, eight of the top ten most-cited journals are also within this region. Such widespread recognition among high-caliber journals underscores the significance and rigor of research endeavors within this specialized area of study. As depicted in **Figure 4**, journals published in the “Molecular Biology Immunology” category and those in the “Medicine Medical Clinical” category are frequently cited by journals in the “Medicine Medical Clinical” category. This indicates that the findings of basic research on macrophages in NSCLC can be translated into clinical practice.

Examining the countries represented in the published literature, major contributors include China, the United States, Japan, Germany, and France (**Figure 5**). Despite China’s leading publication output, collaboration between the United States and other countries appears to be the most extensive. he United States’ strong research infrastructure, funding opportunities, diverse talent pool, interdisciplinary environment, and established international partnerships attract global researchers for collaborative, cutting-edge projects. As result, research outputs originating from collaborations involving the United States are often characterized by their breadth, depth, and global impact within the scientific community. Despite China’s leading position in publications, a considerable amount of highly cited literature does not originate from the country. Immune Thus, fostering global collaborations is crucial for China to expand its influence, driving research advancements, benefiting the global scientific community, and fostering innovation and sustainability.

### 4.2 Emerging macrophage research hotspots in NSCLC: future perspectives

Among the top ten most-cited articles, four primarily focus on immunotherapy and immune response. These studies delve into the relationship between macrophages and immunotherapy in non-small cell lung cancer (NSCLC), including investigating how autologous tumor vaccines enhance T-cell immunity against tumor-associated antigens like HER-2/neu protein when combined with granulocyte-macrophage colony-stimulating factor as an adjuvant (27). Of particular note, some patients experience tumor growth acceleration and worsening of their condition after receiving PD-(L)1 inhibitor therapy, a phenomenon known as tumor hyperprogression (HP) (32). Professor Lo Russo’s research suggests that the reprogramming of macrophages is a significant mechanism underlying HP in NSCLC. Following immunotherapy, tissue samples from all HP patients exhibited tumor infiltration by M2-like CD163+, CD33+, PD-L1+ luster-forming epithelioid macrophages and enrichment of TAM. This indicates the crucial role of TAM reprogramming in inducing HP when combined with immune checkpoint inhibitors (ICIs) and Fc receptor binding, ultimately providing clues to a unique immune phenotype that may predict HP (33).Additionally, four articles focus on Tumor Microenvironment and Cellular Biology. This category encompasses research that investigates the complex interactions between the tumor microenvironment and cellular pathways in NSCLC. Professor Casanova-acebes’ research highlights the role of tissue-resident macrophages in early lung cancer and identifies them as targets for preventing and treating early lung cancer lesions. During early tumor formation, tissue-resident macrophages accumulate near tumor cells, promoting epithelial-mesenchymal transition and invasiveness. They also induce effective regulatory T cell responses, shielding tumor cells from adaptive immunity. Depletion of tissue-resident macrophages reduces regulatory T cell numbers, alters phenotype, enhances CD8 T cell accumulation, and decreases tumor invasiveness and growth. As tumors progress, tissue-resident macrophages redistribute within the tumor microenvironment (TME), with monocyte-derived macrophages predominating in both mouse and human NSCLC (5). These findings underscore the diverse approaches and therapeutic avenues being explored to understand and combat NSCLC, notably the role of macrophages in treating non-small cell lung cancer. From investigations into immunotherapy and immune response mechanisms, including the development of novel vaccines and exploration of immune checkpoint blockade, to studies elucidating the complex interactions within the tumor microenvironment and cellular pathways involving macrophages, researchers are continuously uncovering new insights and potential treatment strategies for NSCLC.

The timeline and temporal zone charts of keywords (**Figures 7B, C**) illustrate the evolving research focus in the field over time. The shift in keywords from early representative terms such as “expression,” “angiogenesis,” “activation,” “growth,” and “survival” to later terms like “microenvironment,” “immunotherapy,” “chemotherapy,” “mechanisms,” and “mutations” reflects significant trends in macrophage research within the context of NSCLC. These trends include: a. Shift to the complexity of the tumor microenvironment: Research has begun to emphasize the role of macrophages as key components of the tumor microenvironment, particularly focusing on blocking the STAT3/IL-10 pathway related to IL-10 (**Figure 7C**), which has seen extensive study in recent years (34, 35). b. Focus on therapeutic interventions and clinical applications: With the advancement of immunotherapy and chemotherapy, researchers have started exploring the role of macrophages in these treatments and their impact on therapeutic outcomes, particularly focusing on nivolumab and docetaxel. c. In-depth exploration of molecular mechanisms and genetic mutations: Technological advancements, especially in high-throughput sequencing, have shifted research towards uncovering the specific molecular mechanisms and genetic mutations in macrophages within tumors (36).

The keyword and reference citation burst charts (**Figures 6D**, **7**) help identify emerging research topics gaining widespread attention quickly. Among the top 25 burst references, six are still in the burst phase. Three of these focus on macrophages and the immune microenvironment. Professor Diether Lambrechts’ study used single-cell RNA sequencing to analyze 9,756 bone marrow cells in non-small cell lung cancer (NSCLC), identifying various cell subpopulations. It found that tumor-associated macrophages had upregulated genes regulated by IRF2, IRF7, IRF9, and STAT2, and downregulated genes regulated by Fos/Jun and IRF8, revealing decreased inflammatory response, TNF-α-induced proliferation, and reactive oxygen species production pathways. This highlights the importance of macrophages in the tumor immune environment and the role of specific transcription factors in M2 polarization (37). Professor Mehrdad Rakaee used multiplex immunohistochemistry to study the immune microenvironment markers M1 (HLA-DR/CD68), M2 (CD163/CD68, CD204/CD68), and pan-macrophages (CD68/CK) in 553 primary tumors and 143 metastatic lymph node samples. The results showed that high levels of HLA-DR/CD68 M1, CD204 M2, and CD68 macrophages were independently associated with improved survival rates in NSCLC patients, particularly with HLA-DR/CD68 M1 levels in lymph nodes being an independent positive prognostic indicator (38). Three studies are related to tumor immunotherapy. Professor Sydney R. Gordon’s research found that high expression of PD-1 on TAMs downregulates macrophage phagocytosis. Blocking PD-(L)1 with immunotherapy can also modulate macrophage function, highlighting the close connection between TAMs and immunotherapy (39).

In summary, these findings align with the keyword burst for “Tumor microenvironment, immunotherapy, polarization” shown in **Figure 7D**. This indicates that research on macrophages remains focused on exploring polarization mechanisms, the relationship between macrophages and the TME, and their interaction with immunotherapy. Targeting lung TAMs can significantly enhance the efficacy of conventional therapies and immunotherapies. However, there is still a long way to go in fully understanding the roles and mechanisms of TAMs in lung cancer progression and developing TAM-based immunotherapies to cure cancer. For example, therapies that regulate TAM polarization or disrupt the supportive interactions between TAMs and the TME could provide new avenues for treating NSCLC. Additionally, focusing on macrophage surface markers and polarization states in anti-angiogenic and immunotherapy treatments could offer valuable insights for predicting treatment prognosis. This could potentially improve patient outcomes by making existing therapies more effective. ​By highlighting these research trends, our study offers valuable insights for clinicians and researchers, guiding the integration of macrophage-focused strategies into clinical practice.

### 4.3 Strengths and limitations

We ensured the accuracy and comprehensiveness of our search formula to include as many relevant research articles on the role of macrophages in NSCLC as possible. This study utilized CiteSpace and VOSviewer for bibliometric and visualization analysis, which not only examined past research progress and hotspots but also deepened the understanding of significant research nodes. Additionally, it provided well-founded predictions for future research trends.

However, this study also has certain limitations. Firstly, we only included literature from the WoSCC database, potentially overlooking articles from other databases. Moreover, articles published after the retrieval date may not have been included due to database access restrictions. Bibliometric analysis itself has inherent limitations as it primarily relies on published research. This reliance may lead to the neglect of certain fields or perspectives, especially those that have not been successfully published or were published in less prominent journals. Additionally, bibliometric analysis may favor more accessible or popular research topics, thereby overlooking valuable yet less popular studies. For instance, the upregulation of CD47 and its inhibition of macrophage phagocytosis did not emerge as a hotspot in this study. However, therapies combining CD47 blockade hold significant clinical translational potential in developing more effective treatments for EGFR-mutant NSCLC (40).

## 5 Conclusion

The discussion on TAMs in NSCLC research trends and hotspots underscores the significance of this field, emerging as a critical factor in NSCLC treatment. Over time, TAMs have garnered increasing attention for their role in both tumor development and potential as therapeutic targets. Evidence suggests that modulating TAM behavior may help suppress tumor growth, dissemination, and drug resistance, thereby improving treatment outcomes for patients.

International collaboration is pivotal in advancing NSCLC research, with partnerships like those between China and the United States accelerating progress and benefiting patients worldwide. To build on these insights, future research should focus on several specific questions: a. Mechanisms of TAM Polarization: What are the underlying mechanisms driving TAM polarization? How can these mechanisms be modulated to shift TAMs from a pro-tumor to an anti-tumor phenotype? b. TAM Interaction with Other Immune Cells: How do TAMs interact with other immune cells in the tumor microenvironment, and how do these interactions influence the efficacy of current immunotherapies? c. Biomarkers for TAM Modulation: What are the most reliable biomarkers for monitoring TAM polarization and activity in patients undergoing therapy? Additionally, our analysis points to the potential for new clinical trials aimed at improving NSCLC treatment outcomes through macrophage-targeted strategies. Specifically: a. Clinical Trials on TAM-Targeting Therapies: Trials that investigate the efficacy of drugs or biologics designed to modulate TAM polarization or disrupt their support of tumor growth could provide new therapeutic options for NSCLC patients. b. Combination Therapies: Evaluating the effectiveness of combining TAM-targeting agents with existing treatments, such as anti-angiogenic therapy or checkpoint inhibitors, could yield synergistic effects and enhance overall treatment efficacy. c. Predictive Markers in Clinical Settings**: Developing and validating predictive markers for TAM activity and polarization states in clinical settings could improve patient stratification and treatment personalization. The role of TAMs in immunotherapy, such as anti-PD-(L)1 therapy, requires further investigation, including how to reprogram macrophages towards anti-tumor activity, modulate the immune microenvironment to induce synergy between macrophages and other immune cells in anti-tumor responses, and how to block macrophage-mediated resistance in NSCLC treatment to enhance therapeutic efficacy. These are expected to become future research hotspots.

Translating laboratory findings into clinical practice and validating TAM-targeted therapies through clinical trials will be crucial for improving survival rates and quality of life for NSCLC patients. By proposing these specific future research directions and potential clinical trials, we aim to guide the integration of macrophage-focused strategies into clinical practice, ultimately enhancing therapeutic outcomes for NSCLC patients. Thus, future research endeavors hold promise for bringing new hope and breakthroughs in NSCLC treatment.

## Data availability statement

The datasets presented in this study can be found in online repositories. The names of the repository/repositories and accession number(s) can be found in the article/Supplementary Material.

## Author contributions

YZ: Data curation, Methodology, Software, Supervision, Visualization, Writing – original draft, Writing – review & editing. TW: Writing – original draft, Writing – review & editing. JS: Writing – review & editing. HB: Writing – review & editing. YX: Writing – review & editing. HW: Writing – review & editing.
